# Monthly Summary

**Published:** 1868-05

**Authors:** 


### MONTHLY SUMMARY.

*How to Extract Silver from Lead.*—To extract silver from lead is very simple; it admits of but one principle, which depends upon the different properties of the two metals. It is a property of lead, when melted in the open air, to lose its metallic appearance, and to burn away into an oxyd of an earthy appearance; on the other hand, it is an essential property of silver not to burn or lose its metallic appearance, when exposed to the action of the strongest fire in the open air. If a mass of metal, consisting of lead and silver, is melted thus, the lead will burn to ashes, or into hard masses of a scaly texture, which mass is known as litharge, while the silver will sink to the bottom of the vessel in which the mass is melted. In practice, where the operation is conducted on a large scale, the silver is extracted from the lead by the oxydation of the lead into a reverberatory furnace of a particular construction. A shallow vessel, called a cupel, is filled with ashes well packed and pounded down, and a cavity cut out for the reception of the nozzle of a bellows, through which air is forcibly driven. When the fire is lighted and the lead in a state of fusion from the reverberation of the flame, the blast from the bellows is made to play forcibly on the surface, and in a short time a crust of oxyd of lead or litharge is formed and driven off to the side of the cupel opposite to the mouth of the bellows, where a shallow aperture is made for it to pass over, another crust of litharge is formed and driven off, and this is repeated until nearly all the lead has been scorified and blown aside. The complete separation of lead is indicated by the appearance of a brilliant lustre on the convex surface of the melted mass in the cupel, which is occasioned by the removal of the last crust of litharge which covered the silver. The silver thus extracted is not sufficiently pure, it is further refined in a reverberatory furnace, being placed in a cupel lined with bone ashes and exposed to an intense heat, so that the lead which escaped oxydation by the first process is converted into litharge, and is absorbed by the ashes of the cupel.—*American Artisan.*

*On the Characteristics of the Different Varieties of Creosote.*—Although the creosote of coal-oil is often employed in pharmacy as a substitute for the creosote of beech-wood, it by no means follows that the two products are identical. An observation made by M. Rust has led to the discovery of a reaction which permits of easily distinguishing between the two creosotes, namely, that creosote from coal forms with collodion a kind of jelly, whilst beech-wood creosote produces nothing of the kind.

In Germany, a mixture of this sort is frequently employed for toothache, composed of equal parts of collodion and creosote: it should be gelatinous. Occasionally the product refuses to gelatinize, and this happens in dealing with beechwood creosote; the collodion thickens a little without loosing fluidity.

The following are other reactions which serve to assist in characterizing the two species of creosote:—

Beechwood creosote is insoluble in ammonia, and gives with excess of weak potash a turbid solution.

Creosote from coal is insoluble in ammonia in the cold, but dissolves, forming a clear solution upon application of heat. It also gives a clear solution with weak liquor potasse.

Lastly, with a neutral solution of perchloride of iron, beechwood creosote, dissolved in alcohol, gives a *green* coloration, whilst an alcoholic solution of the coal-oil is colored *brown*. With aqueous solutions, on the contrary, creosote does not change color, whilst coal-tar creosote furnishes a blue.

According to an observation of M. Hlaiswetz, true creosote is found among the products of the distillation of guiacum resin mixed with a homologue, $C_{14}HgO_4$, which has been named guaiacol. It has been remarked by M. Gorup-Besanez that this latter body is also contained in ordinary wood-creosote in considerable proportion. It is the opinion of several chemists that both these compounds possess the constitution of compound ethers: thus guaiacol is described as oxyphenate of methyl. The empirical formula of creosote is represented as $C_{16},H_{12},O_4$.

M. Frisch confirms, in general, the results obtained by M. Gorup. The creosote upon which he operated was a strongly refracting liquid, of the specific gravity of 1.0784. It was perfectly soluble in alcohol, ether, bisulphide of carbon, as well as acetic acid. With ammonia, which it dissolved slightly, it gave a bluish-green coloration, which changed to brown. It dissolved oxalic acid and several alkaloids. With potash it gradually became brown, and, on addition of ether, developed a blue tint, which disappeared on agitation. It boiled steadily at 195° C. (383° F.). With nitric acid it gave pieriç acid; with hydrochloric acid and chlorate of potash there are formed nacreous scales, consisting of chloranile and birchloroquinone, With sulphuric acid and application of heat it gave, after neutralization with baryta, sulphosalt,—the sulphophenisate of baryta of Laurent,—soluble in alcohol and crystallizable. In the presence of birchromate of potash this sulphophenisic acid acquires an agreeable aromantic odor.

This creosote also answered to the test, alluded to above, by giving with perchloride of iron in alcoholic solution a green color.—*Journ. de Pharm. et de Chimie.*

---

*To Make Linen Fire-Proof.*—A quantity of phosphoric acid lime is dissolved in water; to this a little ammonia is added, and the whole filtered and decolored with animal carbon. It is then put on the fire and left to evaporate until it is concentrated, when gelatine and five per cent. silicic acid is added, and again reduced by evaporation to a crystallic substance, which is dried and pulverized. This powder is called "Hottina," from the name of the inventor. The cloth to be made fire-proof is dipped in a solution made of thirty per cent. of the above powder, thirty-five per cent. of gum, and thirty-five per cent. of starch. The cloth, when dry, will be perfectly fire-proof and preserve its color.—*Druggists' Circular.*

---

*Antidote for Strychnia.*—Dr. J. Bartlett strongly recommends common salt as a curative of strychnia poisoning. He reports as many as twenty experiments on dogs, in which violent symptoms following large doses of strychnia ceased after emetics, induced after drenching the animal with water holding in solution several handfulls of salt.—*Chicago Medical Times.*

---

*Excision of Breast; Anaesthesia by Nitrous Oxide Gas.*—Dr. H. J. Bigelow.—Female, aged 52, had lancinating pains about and in her breast for eleven months. Ten months ago, a small, hard tumour appeared just outside the nipple, and has steadily increased until the present time, when it occupies nearly the whole gland. It is hard, not adherent to the muscle beneath nor the integument above, excepting a space about two inches in diameter in the vicinity of the nipple, which is retracted. No enlargement of axillary glands. Patient, being placed upon the operating table, was made to inspire nitrous oxide gas through a three-valved inhaler, two valves of which were self-acting, one communicating with the gasometer and the other with the atmosphere of the apartment for expiration. The third was controlled by the administrator, L. D. Shepard, D.D.S., by which atmospheric air was admitted when deemed necessary. After a few inspirations the patient became insensible, and in two and a half minutes the operation was commenced by Dr. H. J. Bigelow, and the breast was excised. Nineteen minutes after the inhalation of the gas was begun, the operation was completed and the wound dressed, when the administration was discontinued. In a few minutes, the patient regained her senses, and replied to questions. During the operation, the patient made frequent outcries, and at no time were the muscles relaxed. The face did not exhibit marked lividity, but the blood that escaped from the arteries was of inky blackness. Four hours after the operation, the patient testified that she knew nothing of it. No nausea or other unpleasant symptom supervened. "There was some lividity of the face, but the striking feature of the operation was, what has been well termed in the above account, the inky blackness of the blood, a color far darker than I have ever seen in blood under any circumstances, and altogether darker than occurs in asphyxia from inhalation of ether, where the face is much more livid. Other noticeable circumstances were, the muscular rigidity and the incessant moaning of the patient, who, however, as it afterwards proved, knew nothing of the operation. Altogether, the insensibility, though complete, was far from tranquil or agreeable. But there was no subsequent vomiting."—*Boston Medical and Surgical Journal.*

---

*Sulphuric Acid in Living Animals.*—M. de Luca has announced to the French Academy of Sciences the discovery of free sulphuric acid, to the extent of 3 per cent. in the liquid of some living mollusca. In view of this, his statement, that these creatures when plunged in water evolve carbonic acid, is rather curious.

---

*Ozone.*—Some doubt has been expressed as to the identity of the body in the atmosphere which decomposes iodide of potassium with ozone. Dr. Andrews, of Belfast, long known as a distinguished observer in this direction, has taken up this subject. The substances which may produce this same effect are nitric acid and chlorine, and as it is not impossible that they may exist in the atmosphere, it has been said that we have no certainty of the presence of ozone in the air. Dr. Andrews has shown that heat decomposes ozone so that it produces none of its characteristic effects, after having been subjected to a temperature of 260° C. He found that air which tinged test paper in a tube in two or three minutes, when passing at the rate of three litres in a minute, no longer produced any result when heated to 260° before passing over the test papers. This leaves no doubt that the agent which affects the test paper is really ozone.

---

*Absinthism.*—Such is the title given by a recent French writer to a disease resulting from the excessive use of that favorite *liqueur* absinthe. The dominant character of the new disease appears to be a succession of violent attacks of epilepsy in subjects not otherwise liable to the disease. This is a fair illustration of the modern rage for novelties. Can there be any physician who has had many cases of *mania a portu* to treat without seeing instances of epileptic fits occurring as a substitute for insanity in this disease? Alcoholism from whatever agent can produce this, as well as that from absinthe.

---

*Causation of Phthisis.*—Dr. Bowditch has published an address which he delivered before the Massachusetts Medical Society, and in which he advocates the doctrine that consumption depends largely upon dampness of the soil. He has shown that those portions of New England which are free from this scourge, are characterized by dryness of the soil, but those which he terms "consumption-breeding" districts are remarkable for the dampness of the earth.

*Digitalis.*—Dr. J. D. Brown relates in the *Medical Times*, some remarkable cases of the use of Digitalis in the suppression of the function of the kidneys. He says:

"The rules of management must depend on the pulse. I have seen no good results till the pulse fell in number; it matters not from what figure: fall it must before any change occurs. I would strongly advise 60 as a standard from a high number; 40 or 50 from a lower figure—say from 80. Judging from the effects on the circulation, we cannot lose sight of the fact that the arrest of secretion depends on the capillary congestion, which in turn might, by pressure, paralyze the nerves. The fact, however, remains that we compel the kidney to resume its functions by diminishing the force of the circulation, lessening the quantity of blood by allowing a much longer interval between each new arrival. Strange, too, it is that in four cases the attack commenced suddenly like a fit of stone, and, in reality, stone came away in each case.

"It is not supposed that it will succeed in all cases of that mysterious disease; but it is clear that it has a powerful influence over the renal secretions, and if carefully watched, taking the pulse as a guide, no mischief need be feared."

---

*Tumors of the Tongue and Pharynx.*—The Paris correspondent of the *Medical Record* communicates the following:

M. Desgranges publishes in the *Journal de Lyons*, certain considerations on tumors of the tongue and pharynx, and a special method for operating upon them. This method belongs to M. Sedillot, and consists of a section of the lower maxilla on the median line, by means of which the two halves of the bone could be drawn aside and sufficient space left to excise the tumor. The wound of the soft parts heals readily, but for the cicatrization of the seguments of the maxilla it was found necessary to maintain the adjustment by means of pincers. This instrument presents certain inconveniences, and M. Desgranges has used metallic sutures instead, piercing the bone with a drill, for the passage of the silver wire.

"Two cases are related where this operation was successfully performed for an epithelial cancer of the floor of the mouth. In the first case, the tumor, situated under the tongue, extended from the first molar of the left side to the canine at the right. The posterior face of the maxilla was invaded, and the incisors and left canine were partially loosened from the alveoli.

"In operating, the integuments were divided as far as the hyoid bone, then the section of the maxilla effected by the chain saw. Care was taken that the section should be made at the left side, and the insertions of the genio-hyoid and genio-glossal muscles of the right side avoided. Upon separating the segments of the bone, the diseased parts were easily removed with curved scissors, without touching the subjacent muscles. No blood fell into the pharynx, so that suffocation was avoided. The results were most happy. The tongue retained its movements, and no trouble occurred in the respiration. The two halves of the maxilla were not displaced, and when the patient left the hospital three weeks after the operation, a fibrous callus united the segments, and with sufficient solidity to permit movements of the entire jaw.

"In the second case, the tumor had burrowed more deeply, and was ulcerated. The superficial layers of muscles were removed, but enough remained to insure the movements of the tongue. The operation, performed exactly as in the preceding case, was followed by a slight attack of erysipelas, and it was a month before the two halves of the divided maxilla ceased to shake in the movements of the lower jaw. But in six weeks the osseous union was complete.

"This preliminary osteomy opens a free route to the bistoury; it enables the operator to examine the entire tumor, and to pursue its prolongations, a circumstance essential as a guarantee against relapse. Moreover, the extreme difficulty of ligating numerous arteries encountered in this region is greatly palliated, and finally the danger avoided of suffocation during the anæsthetic sleep, on account of blood flowing into the larynx."—*Medical Gazette.*

---

*Peripheral Termination of the Motor Nerves.*—Prof. TRINCHESE, of the Genoa University, has drawn the following conclusions:—

"1. In all the animals in which he has been able to investigate the subject, a special organ, the *motor plate*, at the end of the *cylinder axis*, has been found.

"2. The following is the manner in which the nervous element is united with the muscular fasciculus:—

"When the muscular fasciculus is provided with sarcolemma and the nervous element with a sheath, this blends with the envélope of the primitive muscular fasciculus at the point at which the nervous element meets with the muscular fascicules. At the same point, or a little before, the medullary substance stops, whilst the *cylinder axis* goes on and enters the motor plate.

"The motor plate is placed beneath the sarcolemma. It appears generally as a cone, of which the summit is directed to the side of the nerve-tube, whilst the base rests on the primitive muscular fibres.

"4. This plate is formed of two superposed and quite distinct layers, especially in those animals which have large plates—the torpedo, for instance. The upper layer is of a granular substance; the lower is perfectly homogeneous, and is probably only an expansion of the cylinder axis.

"5. In the substance of the granular layer of the platc is found, in the torpedo, a system of canals, in which the cylinder axis ramifies as a large-meshed network. These canals are bounded by a sheath which forms their walls.

"6. When the muscular fasciculi have a central canal the granular substance of the plate is prolonged into the granular substance contained in this canal.

"7. In animals provided only with smooth muscular fibres the cylinder axis traverses the granular substance of the plâte dividing into two filaments which have pointed ends at the two extremities of the contractile element.

"8. Altogether it appears that each primitive muscular fasciculus shows one motar plate only. In this one or many nervous elements, proceeding from the subdivision of the same nervous tube, may end.

"9. The diameter of the motar plate increases in proportion with the size of the primitive muscular fasciculus."—*Journ. de l'Anatomie et de la Phys.*, Sept. and Oct., 1867.

---

*Origin and Nature of Movement.*—M. Marey, in a recent work (*Du Mouvement dans les Fonctions de la Vie*), says that physiologists and histologists have been baffled in their attempts to trace the "*origin*" of "*movement,*" or of "contractility" to one single constant anatomical element in the tissues by which this function is manifested. Microscopic observation shows the existence of the power of contraction in tissues which are perfectly *amorphous*, as well as in those that are highly organized. Many of the lower animals—the Medusæ, for example—are eminently contractile, but no special tissue, such as is the seat of contractile power in the higher animals, can be found in them. The Amœbæ also seem composed of a contractile material, in virtue of which they are capable of assuming the most irregular and varied forms, yet the highest powers of the microscope can discover no trace of organized tissue in them. The vibratile cilia of certain epithelial cells, though they present a more advanced organization, show nothing in their transparent tissue which will explain the production of the movements which animate them. Equally difficult is it to explain the movements of the spermatozoa.

But this contractile material, notwithstanding its different forms, is similarly affected by physical and chemical agents. Thus heat stimulates its contractility, cold diminishes it. The action of alkalies favours it, that of acid destroys it. It has therefore been supposed that the endowment of contractility resides in a substance chemically the same in all cases, but appearing under different aspects. In opposition to this supposition is the fact that the chemical composition of one muscle will vary with that of another, within certain limits—inosite, for example, is found in the muscles of the heart, while it is wanting in most other muscles. M. Marey is therefore obliged to leave the discussion of the "origin of movement" very much as he found it—unexplained, perhaps inexplicable.—*Med. Times and Gaz.*, Feb. 15, 1868.

---

*Influence exerted by Anœsthetics on the Brain and Nervous System.*—This was the subject of a recent lecture by Dr. B. W. Richardson. The obvious fact that the motion of the heart and the movements of respiration continue in action while the rest of the body is under the narcotic effect, during anæsthesia, proves that the whole nervous system is not involved, and that the involuntary and semi-voluntary muscular mechanism is also not involved except when extreme and fatal symptoms are developed. What parts, then, are influenced by an anæsthetic? The idea was almost intuitive that the brain is the organ affected, and that the centres of consciousness are those chiefly held in abeyance. But, to prove this as true, experiment was necessary. In proof, the lecturer took a large pigeon, narcotized it deeply with chloroform, and in this state passed through its body, from the head to the foot, a rapid intermittent induction current. The bird instantly rose from the table, extended its wings, opened its eyes, and seemed as if restored; the current was then stopped, and the bird was shown to be as deeply asleep and as powerless as before. Another bird was put to sleep by freezing the brain, and when utterly insensible was subjected to the electrical shock in the same way, when it flew from the table into the room, where, breaking its connection with the battery, it dropped on the floor comatose, motionless, and as anæsthetized as before, in which condition it remained for many minutes. The lecturer in these experiments demonstrated that the anæsthetic action was localized in the cerebrum. His battery was like an outer brain, which supplied power without intelligence, and which, by the effects of its current, showed that all the muscular elements were ready for work, and only awaited the order from the brain. The lecturer next discussed the question—What, during the process of anæsthesia, leads to this change in the brain? Is there a chemical action on albumen? Is there pressure on brain matter? Is there deficient oxidation of the blood? Is there contraction of bloodvessels, and diminished supply of blood from that cause? All these hypotheses were experimentally tested and negatived. It was admitted that during extreme anæsthesia there is reduced oxidation and a singular reduction of temperature. These changes are inevitable, because the anæsthetic vapours replace oxygen during their diffusion into blood; but the diminished oxidation is not the cause of the insensibility. In proof of this Dr. Richardson showed an animal breathing an air in which the oxygen was reduced by addition of nitrogen from 21 parts to 9 parts in the 100, side by side with another similar animal breathing an air in which the oxygen was reduced by the addition of vapour of bichloride of methylene only to about 20 parts in the 100—viz., 4 cubic inches in 500. The result was that the animal in the extremely reduced atmosphere was quite unaffected whilst the animal in the slightly reduced atmosphere was in the deepest narcotism. Then a correcting experimental test was adopted, and the bichloride was administered in an atmosphere containing an excess of oxygen, the oxygen being present in double its ordinary or natural proportion; the excess of oxygen exerted no perceptible obstacle to the anæsthesia. To determine whether there was contraction of bloodvessels under anæsthetics, the lecturer had had recourse to transparent small trout; through their bodies, with the microscope and the one-inch lens, the bloodvessels could be seen, and the corpuscles flowing through them. These animals can be narcotized readily by making them breathe water saturated with chloride of methylene or ether. In the narcotized condition, the vessels do not contract, but under the influence of ether, in the later stages before death occurs, dilatation and regurgitation are observed. The latter is noticed also when chloride of methylene is used. With both reagents breathing and vessel circulation cease before the heart's action. The lecturer concluded that anæsthetic vapours act directly upon nerve matter either by preventing the development of force or by stopping conduction. The latter hypothesis is supported by the fact, proved by experiment, that these vapours obstruct the conduction of heat and electricity.—*Med. Times and Gaz.*, Feb. 15, 1868.

---

*Influence of Electricity on Muscular Fibre of Vegetable Life and on Nutrition.*—M. Onimus has observed the following facts in relation to this subject. 1. *Action on the great sympathetic.* The intermittent current produces a lowering of the temperature; the continued current, on the contrary, raises it. With the intermittent current there is a tetanic spasmodic contraction of all the muscular fibres of the small bloodvessels, preventing the flow of blood. There are no corresponding effects with the continued current. 2. *Action on the intestine.* The action of the intermittent current is local; the electrified part of the intestine becomes violently contracted. The continued current produces no effect. 3. *Action on the heart.* When applied directly to the heart of cold-blooded animals, the intermittent current produces two or three contractions; the movements of the heart cease, and the auricle remains forcibly contracted.

The continued current does not arrest the pulsations of the heart, the movements, on the contrary, are more frequent.—*Brit. Med. Journ.*, Jan. 25, 1868, from *Archives Gen. de Med.*

---

*New Method of Local Anaesthesia, Applicable to the Extraction of Teeth and other Operations in the Cavity of the Mouth.*— The following communication by Henoque and E. Fredel deserves the fullest attention of surgeons and dentists. It opens a new means for the painless performance of operations in the mouth, without inducing general anæsthesia, or submitting to the numerous inconveniences of Richardson's method when applied to these parts. The attention of the profession having been particularly called to this subject by Magitot's representation of the disadvantages of local anæsthesia as hitherto applied to operations in the mouth, two physicians of Paris, Henoque and Fredel, inaugurated the attempt to produce the anæsthesia required by the application of the ether spray along the course of the trifacial nerve outside of the mouth. The well known practice of introducing narcotic substances, chloroform liniments, etc., into the external auditory canal for the purpose of relieving toothache, induced the experimenters to test the value of the anæsthetization of this canal as a means to the painless removal of teeth. Anæsthesia of the meatus auditorius externus was accomplished by the use of ether, with the ordinary atomizing tubes, and the results were as follows :

Of thirty-two individuals who had teeth extracted, twenty-four experienced no pain whatever; in five cases the extraction was painful, and in three cases the result was doubtful.

Among the cases where the absence of pain was complete were five extractions of upper molars, and one of a canine tooth; among the painful cases were three upper molars and one lower bicuspid.

It only remains to establish a sure criterion of the full accomplishment of anæsthesia. In the cases reported by Henoque and Fredel, it appeared necessary to continue the application of the ether spray to the external meatus for at least three minutes. These gentlemen believe that the procedure above described is applicable to other operations in the mouth. They have employed it in one case for the removal of the tonsils, and the patient, an intelligent young man, declared that he experienced not the slightest pain. They commend their method to the further trial of the profession.—*Gaz. Hebdom.*—*Aerz'liches Literaturblatt. Med. Gazette.*

### BIBLIOGRAPHICAL NOTICES.

